# Evaluation of a new rapid diagnostic test, based on the chimeric protein Q5, for the diagnosis of human and canine forms of visceral leishmaniasis

**DOI:** 10.1590/0074-02760250126

**Published:** 2026-05-11

**Authors:** Wagner José Tenório dos Santos, Natalia Rocha Nadaes, Allana Kelly Oliveira Dutra, Adalucia da Silva, Hemilly Rayanne Ferreira da Silva, Artur Leonel de Castro Neto, Virgínia Maria Barros de Lorena, Valeria Rêgo Alves Pereira, Allana Maria de Souza Pereira, Maria Edileuza Felinto de Brito, Milena de Paiva Cavalcanti, Zulma Maria Medeiros, Kamily Fagundes Pussi, Herintha Coeto Neitzke-Abreu, Valeria Marçal Felix de Lima, Carlos Henrique Nery Costa, Keila Gisele Azevedo F dos Santos, Edimilson Domingos da Silva, Osvaldo Pompilio de Melo Neto

**Affiliations:** 1 Fundação Oswaldo Cruz-Fiocruz, Instituto de Tecnologia em Imunobiológicos, Bio-Manguinhos, Rio de Janeiro, RJ, Brasil; 2 Fundação Oswaldo Cruz-Fiocruz, Instituto Aggeu Magalhães, Recife, PE, Brasil; 3 Universidade Federal de Grande Dourados, Dourados, MS, Brasil; 4 Universidade Estadual Paulista, Araçatuba, SP, Brasil; 5 Universidade Federal do Piauí, Teresina, PI, Brasil

**Keywords:** RDT, ELISA, visceral leishmaniasis, recombinant protein

## Abstract

**BACKGROUND:**

Visceral leishmaniasis (VL), caused by *Leishmania infantum*, is the most severe form of leishmaniasis, prevalent in many countries, but still with limitations in diagnosis for both human and canine hosts. Serological assays based on recombinant proteins are the most efficient diagnostic alternatives, with the rapid diagnostic test (RDT) being the most cost-effective. The previously described chimeric Q5 is a recombinant protein derived from three native *L. infantum* antigens, which is potentially useful for both human and canine VL diagnosis, through preliminary enzyme-linked immunosorbent assay (ELISA), but which was not evaluated within an RDT setting.

**OBJECTIVES:**

To evaluate the diagnostic performance of the chimeric recombinant protein Q5 in both ELISA and RDT formats for the detection of human and canine VL, and to compare its performance with RDTs based on Lci2 and Lci13 antigens.

**METHODS:**

Here, we first expanded the Q5 evaluation through ELISA with a larger set of human and canine VL-positive sera from multiple origins. We confirmed a sensitivity ranging between 80% and 90% for the human VL and greater than 90% with the canine VL sera. A new RDT-Q5 was then set up and tested with multiple batches of human and canine sera.

**FINDINGS:**

An improved performance was seen for the human VL diagnosis (94% sensitivity), but it was reduced with canine sera (86% sensitivity). Specificity values for both the ELISA-Q5 and RDT-Q5 were generally greater than 95%, with limited (8%) or no false-positive results with human sera from individuals with cutaneous leishmaniasis (CL) and Chagas disease (CD), respectively. The RDT-Q5 performance was compared with RDTs based on two other recombinant proteins, Lci2 and Lci13, tested respectively for the human and canine VL diagnosis.

**MAIN CONCLUSIONS:**

Despite an equivalent performance for the human VL diagnosis, the RDT-Lci2 led to a much greater incidence of false-positive results with the CL and CD sera. In contrast, no setup for the RDT-Lci13 was effective with the canine sera. Our results confirm the RDT-Q5 as an efficient alternative for the VL diagnosis in the field, particularly for the human form of the disease.

Visceral leishmaniasis (VL) is the most severe form of leishmaniasis, still prevalent in many developing countries and frequently associated with high morbidity and mortality cases. The protozoan *Leishmania infantum* is the agent responsible for VL in Latin America, where VL is considered a zoonosis, with animal hosts including domestic dogs and, less frequently, other mammals.^1^ ^,^ ^2^ The World Health Organisation (WHO) included VL as a neglected tropical disease, requiring, as targets for 2030, the development of new tools for its prevention, diagnosis, and treatment. Sixty-five of the 70 countries where VL is endemic have been validated for possible VL elimination as a public health problem by 2030. These include Brazil, Ethiopia, India, Kenya, Somalia, South Sudan, and Sudan, where more than 90% of the VL cases reported worldwide are found.^3^ The current context shows the need for developing more sensitive and rapid diagnostic tests for early VL detection in humans as well as in animal reservoirs, particularly dogs, to prevent its spread.

Parasitological diagnosis is considered the gold standard for the identification of hosts infected with *Leishmania*. It requires invasive collection procedures, however, and it is time-consuming, with sensitivity depending on the presence of parasites in the collected samples. Various serological assays have been developed for VL diagnosis, based on the detection in serum or blood samples of antibodies, mainly IgG and IgM, against *Leishmania* proteins. These assays are generally restricted to specific hosts, being dependent on the use of secondary antibodies, such as anti-human or anti-canine IgG, conjugated to an enzyme or fluorescent molecule, for easy detection. Assays available for VL diagnosis include the indirect fluorescent antibody test (IFAT), the enzyme-linked immunosorbent assay (ELISA), the immunoblotting, and the immunochromatographic (IC) or rapid diagnostic test (RDT).^4^ ^,^ ^5^ ^,^ ^6^ ^,^ ^7^ ^,^ ^8^ ^,^ ^9^

Serological diagnostic assays based on recombinant proteins, as compared to similar assays using crude or whole *Leishmania* antigens, are easier to produce, cost-effective, and can potentially lead to lower cross-reactions with VL-related diseases.^3^ ^,^ ^5^ ^,^ ^10^ ^,^ ^11^ Most used assays for human VL diagnosis are based on the rK39 recombinant antigen, derived from a *L. infantum* kinesin,^12^ but although the commercially available RDTs based on rK39 were early on seen to be very efficient in the Indian subcontinent, a lower performance was reported elsewhere.^13^ ^,^ ^14^ ^,^ ^15^ A chimeric recombinant antigen, rK28, consisting of fragments from three different *Leishmania* antigens, including rK39, has been subsequently produced and found to have a superior diagnostic performance through both ELISA and RDTs with sera from Sudan.^16^ ^,^ ^17^ Another kinesin derivative, more recently developed, found to have a very significant performance for the human VL diagnosis is the recombinant kDDR.^18^ Despite the available antigens, however, current strategies for large-scale and cost-efficient VL diagnosis would still benefit significantly from the identification of alternative recombinant antigens with possibly greater efficiency under less favourable conditions, such as in asymptomatic or immunocompromised individuals.

Rapid tests based on rK39 have also been evaluated for the canine VL diagnosis but have been found with variable or suboptimal performances.^6^ More efficient results were seen with a synthetic protein derived from the fusion of rK39 with the rK26 antigen, derived from a hydrophilic repetitive protein.^19^ This antigen was used to develop a Dual-Path Platform (DPP) test,^20^ which is currently recommended for the VL diagnosis in dogs in Brazil. This assay has been assessed through several studies, which confirm its usefulness but highlight the need for further improvements in performance.^21^ ^,^ ^22^ ^,^ ^23^ ^,^ ^24^ ^,^ ^25^ Many other recombinant antigens have also been evaluated for the canine diagnosis, mostly through ELISA assays but, despite promising results, few have been thoroughly investigated or evaluated as the basis for rapid IC tests.^6^ ^,^ ^11^ ^,^ ^18^ ^,^ ^26^ ^,^ ^27^ ^,^ ^28^

Serological assays optimised with the use of antibody-binding proteins, such as protein A (from *Staphylococcus aureus*), can potentially be used for the VL diagnosis from multiple hosts. Protein A exhibits high affinity for several classes and subclasses of antibodies, such as IgG (mostly) and IgM, from several species, including humans, rabbits, and dogs, despite having only a weak interaction with bovine and mouse antibodies.^29^ To be effective, however, such assays require recombinant *Leishmania* antigens that are efficiently recognised by antibodies from multiple species. This is a limiting condition, also observed in previous studies by some of us, which identified several new recombinant antigens potentially useful for the serological VL diagnosis. Best antigens for human VL diagnosis, such as Lci2, derived from the same gene encoding rK39, did not perform well with the canine sera. In contrast, those with the best performance for dogs, including Lci3, Lci12, and Lci13, were not efficient with the human sera.^30^ ^,^ ^31^ To produce a single recombinant protein potentially useful for the diagnosis of both human and canine VL, fragments derived from three of the best antigens previously identified (Lci2, Lci3, and Lci12) were joined into new chimeric polypeptides. These were then evaluated through ELISA assays regarding their potential use for VL serodiagnosis with human and canine sera. The best chimeric protein (PQ), named Q5, showed efficient sensitivity results in sera from humans (82%, N = 50) and dogs (100%, N = 39), with no positive reactions with healthy control samples.^32^

In the present study, the recombinant Q5 protein was evaluated using a larger panel of human and canine sera through separate ELISA assays, specific for each host, as well as a protein A-based RDT, applicable to both humans and dogs. The ELISA-Q5 confirmed the efficient performance seen previously with both human and canine VL, while the Q5-based RDT demonstrated equivalent or superior sensitivity and specificity compared to current diagnostic tests for both hosts. Based on these findings, we hypothesise that combining the Q5-based RDT with the ELISA may further enhance diagnostic robustness. The use of RDT-Q5 as a screening test with the rELISA-Q5 having a confirmatory role, for example, may provide a complementary approach that improves accuracy, particularly in diverse clinical settings or in cases with borderline results.

## MATERIAL AND METHODS

**Fig. 1:**
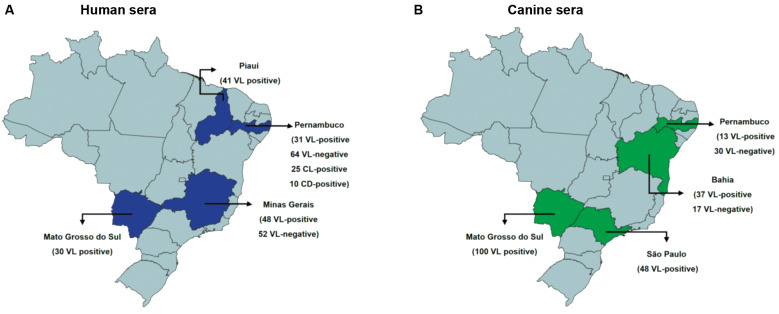
description of human and canine serum panels employed in this study: geographic distribution in Brazil, highlighting the states where human (blue) and canine (green) serum samples were collected. These samples were used throughout the study, except for the visceral leishmaniasis (VL)-positive and VL-negative human sera from Minas Gerais (MG), which were exclusively tested with the rapid diagnostic test (RDT)-Q5 by Enrique Dias Foundation (FUNED)-Laboratório Central (LACEN).

*Human sera and ethical considerations (CAEE)* - A total of 102 human sera with a previously positive VL diagnosis were used in the current study Supplementary data (Table). A first set of 41 positive sera was derived from parasitologically confirmed individuals, from the Brazilian State of Piauí (PI) (Protocol CAEE: 0116/2005). These were previously described and used in the first evaluation of the Q5 protein through ELISA.^32^ Another set of VL-positive sera included 30 sera from the State of Mato Grosso do Sul (MS), all with a polymerase chain reaction (PCR) positive result plus parasitological confirmation or at least one other positive result from an independent serological test, such as the ELISA-rK39, RDT-rK39, IFAT or ELISA with whole parasite extract (their use was ethically approved according to the protocol CAEE55556916.2.0000.5160). Similarly, a third sera set, from Pernambuco (PE) state, includes nine with a positive parasitological test plus 22 additional sera tested through PCR or the serological VL tests described above from MS, with a minimum of two positive results (their use approved by the protocol CAEE 0121.0.095.00-08). A total of 64 negative sera from the PE State were selected with negative parasitological tests and negative results in at least two of three serological tests (ELISA-rK39, RDT-rK39, ELISA with soluble parasite protein). Sera from individuals with confirmed cutaneous leishmaniasis (CL) (25 sera) or Chagas disease (CD) (10 sera) were also used. To complete the studies, a last batch of sera was evaluated by the Enrique Dias Foundation (FUNED) from the state of Minas Gerais (MG) (48 positive and 52 negative sera) (Fig. 1A).

*Canine sera and ethical considerations* - A total of 185 VL-positive canine sera Supplementary data (Table), all from parasitologically confirmed dogs, were used (Fig. 1B): 100 from the MS State (Protocol: Nº27/2016); 48 from the State of São Paulo (SP) (Protocol: Nº16/2014); and 37 from the State of Bahia (BA) (Protocol: Nº40/2005), these also used in the preliminary assessment of Q5 through ELISA.^32^ In addition, 13 sera were used from PE (Protocol: N° 76/2014) from dogs with the VL diagnosis confirmed through positive results from at least three independent serological assays: DPP, IFAT, and ELISA SLA, based on soluble *Leishmania* antigen. A total of 47 sera were also used, with negative results from both DPP and ELISA SLA: 30 from the PE State, all of which were also negative in the ELISA-rK39; and 17 from the State of BA. The origins of all the sera used here and in the following topics are represented in (Fig. 1A).

*Expression and purification of recombinant proteins* - Expression of the Q5 protein was carried out as previously described,^32^ but with bacterial growth at 37ºC. Protein purification was performed through affinity chromatography with the AKTA Pure (Cytiva) set up, with buffer A (20 mM Tris-HCL, 8 M urea, 20 mM imidazole and 250 mM NaCl pH 8.0) and buffer B (20 mM Tris, 8 M urea, 500 mM imidazole, and 250 mM NaCl pH 8.0), used respectively for washes and elution. The recombinant proteins Lci2 and Lci13 were expressed in *Escherichia coli* (BL21 (DE3) and purified as described in previous studies,^30^ ^,^ ^31^ in the presence of 8 M urea.

*ELISA assays using recombinant proteins (rELISA)* - The methodology used for the ELISA tests was already described,^32^ where a dilution of 1:2500 was used for the human sera, and 1:900 for the dog sera, and the tests were performed with the Q5 protein purified in 8 M urea (6 µg/mL). For the ELISA with the human and canine sera, the peroxidase conjugated goat anti-human IgG (Sigma-Aldrich diluted 1:10.000) or anti-dog IgG (Sigma-Aldrich diluted 1:1.200) was used, respectively.

*RDT based on the recombinant* - For the RDT prototypes, the purified recombinant proteins (Q5, Lci2, or Lci13) were impregnated on nitrocellulose membranes, also impregnated in parallel with the protein A control, following the same parameters of a previous study.^33^ All RDTs tested here were based on the following set-up sequence: sample pad, conjugate pad, nitrocellulose membrane impregnated with the antigen (for the test line) and the control, followed by the sink pad (to absorb residues from the run). These were cut into strips, 5.2 mm wide, and placed in plastic cassettes. After various analyses, the final test conditions for the optimised RDT-Q5 required the impregnation with 0.1 microliter of protein per millimetre of membrane. These tests used protein A for serum detection, and the same tests were used for the sera from both humans and dogs. The test procedures were standardised so that five (serum) or ten (blood) microliters of the sample could be added to each well, followed by the addition of three drops of running buffer to allow the sample fluid to flow through capillary action along the length of the nitrocellulose membrane. The results were determined visually after 20 min of incubation at room temperature (average 22ºC). Positive results were indicated by the appearance of bands coloured pink or purple on the test and control lines, while only the control line appeared for negative results. If the control line did not appear, the test was considered invalid. Tests were then set up for different batches of recombinant proteins. The RDT cassettes were stored at room temperature in silica-protected containers until use.

*Statistical analyses* - Sensitivity, specificity, and confidence interval parameters were estimated with the software MedCalc (version 12.3) (MedCalc Software, Ostend, Belgium). The GraphPad Prism program was used to generate the dot plot, the receiver-operating characteristics (ROC) curve, and the bar chart (GraphPad Prism version 6.00 for Windows, GraphPad Software, La Jolla, California, USA). The cut-off was determined by adding twice the standard deviation of the negative sera optical density to the mean optical density of the negative samples, corresponding to a 95% confidence interval.

## RESULTS

**Fig. 2:**
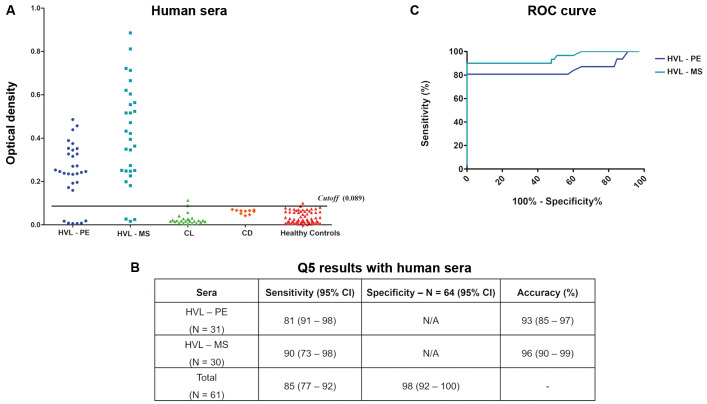
evaluation of the enzyme-linked immunosorbent assay (ELISA)-Q5 protein with human sera. (A) ELISA-Q5 results after testing with the human visceral leishmaniasis (HVL) sera from Pernambuco (PE) and Mato Grosso do Sul (MS). (B) Summary of the ELISA-Q5 results with the defined values for the sensitivity, specificity, and accuracy parameters is shown, with the 95% confidence intervals (CI) indicated in parentheses. (C) Receiver-operating characteristics (ROC) curve analysis based on the ELISA results shown. Each dot in the ELISA assay represents the average of a technical triplicate.

*ELISA-Q5 for the diagnosis of human VL* - Thirty-one sera from PE were first tested, with six producing false negative results and defining a sensitivity of 81%. Another 30 sera from MS were also tested, with three false negative results and 90% sensitivity (cutoff 0.089). To determine specificity, 64 VL-negative sera were also tested, with only one producing a false positive result (Fig. 2A-B). ROC curves were next generated comparing the performance of the ELISA-Q5 with the sera from the two Brazilian states and confirming its greater performance with the sera from MS (Fig. 2C). Combining the data for the entire set of tested sera revealed an overall sensitivity of 85%, with 98% specificity. Next, we tested twenty-five sera from patients with CL, with only two sera testing positive (8%), followed by testing of ten sera from individuals with CD, with no positive results. Overall, these results confirm a very good performance of the ELISA-Q5 for the human VL diagnosis.

**Fig. 3:**
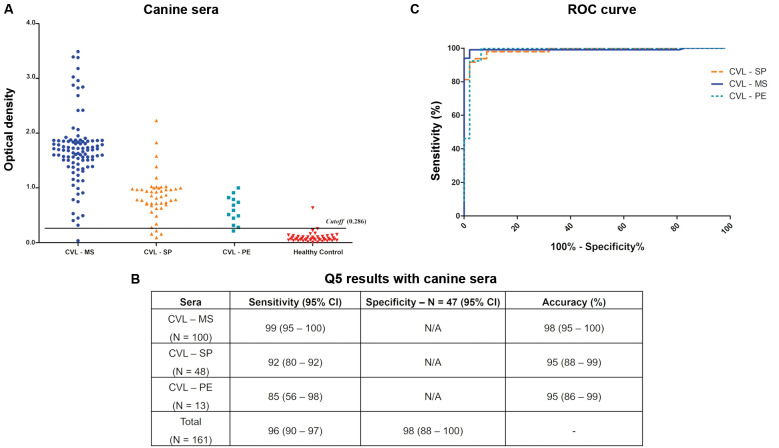
evaluation of the enzyme-linked immunosorbent assay (ELISA)-Q5 protein with canine sera. (A) ELISA-Q5 results after testing with the canine visceral leishmaniasis (CVL) sera from Pernambuco (PE), Mato Grosso do Sul (MS), and São Paulo (SP). (B) Summary of the ELISA-Q5 results with the defined values for sensitivity, specificity, and accuracy parameters is shown, with the 95% confidence intervals (CI) indicated in parentheses. (C) Receiver-operating characteristics (ROC) curve analysis based on the ELISA results shown. Each dot in the ELISA assay represents the average of a technical triplicate.

*ELISA-Q5 for the canine VL diagnosis* - In this study, we reassessed the ELISA-Q5 using a significantly larger sample of VL-positive canine sera (summarised in Fig. 1B). We first tested two sets of parasitologically confirmed sera from two different Brazilian states: 100 sera from MS and 48 sera from SP (all ELISA-Q5 results with the canine sera shown in Fig. 3A-B). For the MS sera, only one negative result was observed, yielding a sensitivity of 99% for the assay. In contrast, the SP sera showed a sensitivity of 92%, with four false-negative results. A third set of VL-positive sera, from PE, included 13 sera diagnosed as positive by both EIE-LVC and DPP tests. Among these, two negative results were recorded with ELISA-Q5, resulting in a sensitivity of 85%. To determine specificity, we also tested 47 sera with a previously defined negative diagnosis, of which only one was a false positive (cutoff 0.286). Separate ROC curves were also generated for all three sera sets, showing the equivalent performance of the ELISA-Q5 with all (Fig. 3C). Overall, based on the 161 positive sera tested, the ELISA-Q5 demonstrated 96% sensitivity and 98% specificity for diagnosing VL in dogs, consistent with the excellent performance originally defined for this assay.

**Fig. 4:**
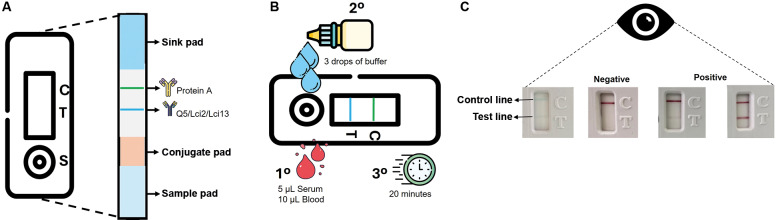
description of the cassettes used for the rapid diagnostic tests (RDTs) evaluated in this study. (A) The assembled strips found within the cassettes included: a card to hold the buffer and red blood cells (named “Sample pad”); the membrane loaded with colloidal gold (“Conjugate pad”); a nitrocellulose membrane where the different antigens assessed (Q5 or Lci2 or Lci13) were loaded (blue line), as well as the protein A (control, green line); and the waste membrane (“Sink pad”). (B) Five microlitres of serum or ten microlitres of human or canine blood were used for each test, carried out with three subsequent drops of running buffer followed by up to 20 min incubation for the results to appear. (C) The test result may indicate reactivity on the “Test line” (negative or positive, strong or weak) or an invalid test (no signal on the Control line). (C) Control line. T: test line; S: sample well.

*RDT based on the recombinant Q5* - Considering the significant ELISA-Q5 performance, we next aimed to evaluate its performance as part of an immunochromatographic RDT test based on a lateral flow platform. The RDT used here, represented in Fig. 4A, allows loading of either sera or blood samples into wells (Fig. 4B), with a positive result indicated by the appearance of bands on the test and control lines (Fig. 4C). To compare the Q5 in the RDT with another recombinant antigen with similar performance in the ELISA assay for the human VL diagnosis, we also set up an experimental RDT prototype based on the Lci2 antigen. Likewise, a third RDT was set up with the recombinant Lci13, aiming for a comparative analysis with the canine sera only.

**Fig. 5:**
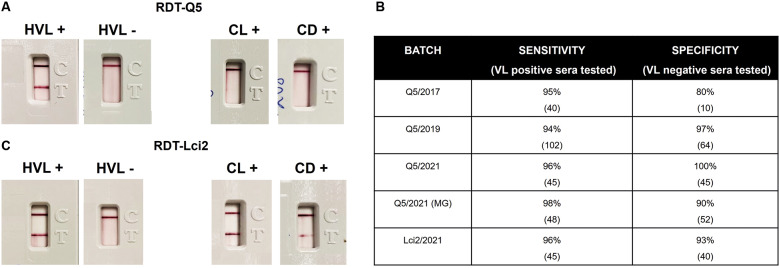
reactivity of the new rapid diagnostic tests (RDTs) described here with human visceral leishmaniasis (HVL) sera. (A) Representative results showing the reactivity of the RDT-Q5 with positive and negative HVL sera, as well as with sera from individuals with cutaneous leishmaniasis (CL) and Chagas disease (CD). The test and control lines are seen in pink/purple. (B) Summary of the results from testing the various batches of RDTs assessed here. (C) Representative results showing the reactivity of the RDT-Lci2 with VL-positive, VL-negative, CL-positive, and CD-positive sera.

*Evaluation of the RDT-Q5 for the human VL diagnosis* - For a first assessment of the RDT-Q5 applied for the human VL diagnosis, a first batch was produced (Q5/2017) and tested, with representative positive and negative results shown in Fig. 5A. The RDT-Q5 was first evaluated with a limited panel of 41 VL-positive human sera from Piaui, with only two of those sera producing a false negative result. Ten negative sera, from Pernambuco, were also tested, with two producing a false positive result, with the preliminary sensitivity and specificity values defined at 95% and 80%, respectively (Fig. 5B). A second batch of the RDT-Q5 test was then produced at similar conditions (Q5/2019) and assessed with a total of 102 VL-positive sera from three Brazilian states (PI, MS and PE), with six producing false negative results (94% sensitivity). A total of 64 sera from negative controls were also tested, but only two sera showed a positive result (97% specificity). This test was also assessed with sera from 25 individuals afflicted with CL and ten with CD (Fig. 5A). Only two of the CL-positive sera produced positive results, indicating low cross-reactivity with the cutaneous form of the disease and no cross-reaction with the CD sera, even with the very effective performance with the human VL sera.

Aiming to improve further the performance of the tests, a third batch was produced with new setup conditions (Q5/2021), which included the recombinant Q5 purified using 2 M urea (all other protein purifications used for the assays described here used 8 M urea). This new batch was evaluated with a selection of 45 VL-positive and 45 VL-negative sera from those tested with the Q5/2019 batch, with only one serum producing a false negative result (96% sensitivity/100% specificity overall). The Q5/2021 batch was also sent to an independent laboratory [FUNED-Laboratório Central (LACEN), from the State of MG], where values of 98% sensitivity (N = 48) and 90% specificity (N = 52) were observed. These results confirm the reproducibility of the RDT tests based on the chimeric Q5 but do not show any substantial improvement with the lower urea concentration.

We next opted to compare the performance of the RDT-Q5 with an equivalent test based on the Lci2 antigen. The RDT-Lci2 was then evaluated with a limited number of sera: 45 sera from VL-positive individuals, 40 sera from negative controls, 10 sera for CL, and 10 sera for CD. The results showed a sensitivity of 96% and a specificity of 93% (Fig. 5B-C), but cross-reactions were seen not only with sera from individuals with CL, with six positive results, but also with CD sera, with four of those producing a positive result. Despite a similar efficiency for the VL-diagnosis with the human sera, when compared to the RDT-Q5, the RDT-Lci2 is therefore less able to avoid cross-reactions or false positive results with the closely related diseases.

**Fig. 6:**
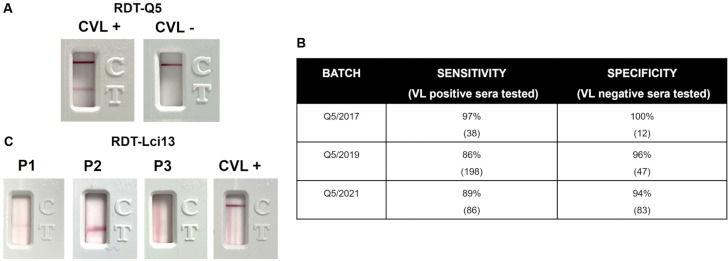
reactivity of the different batches of the rapid diagnostic test (RDT)-Q5 with canine visceral leishmaniasis (CVL) sera. (A) Representative results showing the reactivity of the RDT-Q5 test with positive and negative CVL sera. (B) Summary of the results from the testing of the various batches of the RDT-Q5 assessed here. (C) Representative results showing the reactivity of various setups for the RDT-Lci13. Several of those tested various buffers and protein concentrations, but the test line, or smears, appeared in the absence of the canine VL-positive sera (P1, P2, P3). With other setups, the test line did not appear even after testing with multiple sera from VL-positive dogs (CVL+).

*Evaluation of the RDT-Q5 for the VL diagnosis with canine sera* - All the RDT tests used protein-A for the detection of VL-positive antibodies and could potentially be used not only with human sera but also with sera from different animal species. For a preliminary evaluation of the RDT-Q5 for the canine VL diagnosis, the first batch produced (Q5/2017) was also assessed with a limited panel of 38 sera from confirmed VL-positive dogs, as well as from 12 negative control animals, from Bahia. Representative positive and negative results are shown in Fig. 6A, with only one false-negative and no false-positive results observed, leading to 97% sensitivity and 100% specificity values (Fig. 6B). Another assessment was performed with the second RDT-Q5 batch produced (Q5/2019) and a larger set of 198 VL-positive and 47 VL-negative sera from four Brazilian states, including those sera already assessed with the Q5/2017 batch. The results, however, indicated a poorer performance for the test, with 86% sensitivity and 96% specificity. With the changes made to the third RDT-Q5 batch described above (Q5/2021), yet another evaluation was carried out with a subset of 82 VL-positive and 17 VL-negative canine sera, with observed sensitivity and specificity values of 89% and 94%, respectively (also shown in Fig. 6B). These results are consistent with the less efficient performance of the RDT-Q5 with the canine VL sera, compared to its performance for the human VL diagnosis.

Attempts were also made to properly evaluate the RDT-Lci13 for a comparative assessment regarding the canine VL diagnosis. However, despite multiple optimisation steps and modifications regarding membrane types, protein concentrations, and buffers, none of the setups tested were able to bring any efficiency for this test in identifying VL-positive canine sera (Fig. 6C).

*Comparative analysis of the diagnostic performance of Q5 using ELISA and RDT* - Overall, the results shown so far indicate a better performance for the ELISA-Q5 with the sera from VL-positive dogs, while the RDT-Q5 performed better with the human sera, with specificity values for both tests generally found to be greater than 95%. To fully understand this discrepancy, we opted to directly compare the results from the ELISA-Q5 with the second RDT-Q5 batch (Q5/2019), both tested with larger assemblages of sera from multiple Brazilian states for both human and canine sera, as shown in Table. When the human sera are considered, both tests were assessed with the same set of 102 VL-positive sera, including 41 sera from PI, whose results for the ELISA-Q5 were reported in our previous publication.^32^

**TABLE:** Agreement between the performance of enzyme-linked immunosorbent assay (ELISA)-Q5 and rapid diagnostic test (RDT)-Q5 using visceral leishmaniasis (VL)-positive and human VL (HVL)-negative and canine VL (CVL) sera

		ELISA-Q5 +	ELISA-Q5 -	Total	Agreement
HVL positive	RDT-Q5 +	87	9	96	91,17% (93/102)
	RDT-Q5 -	0	6	6	
	Total	87	15	102	
HVL negative	RDT-Q5 +	0	2	2	95,31% (61/64)
	RDT-Q5 -	1	61	62	
	Total	62	2	64	
CVL positive	RDT-Q5 +	169	1	170	90,91% (180/198)
	RDT-Q5 -	17	11	28	
	Total	186	12	198	
CVL negative	RDT-Q5 +	1	1	2	97,87% (46/47)
	RDT-Q5 -	0	45	45	
	Total	1	46	47	

Number of sera with positive (+) or negative (-) results with either the ELISA-Q5 or the RDT-Q5, with the agreement values (total number of sera with the same results in both tests/total number of sera assessed).

All six sera producing negative results with the RDT-Q5 were also found to be negative with the ELISA-Q5, but the latter test also produced negative results for nine other sera, confirming the reduced performance seen for the ELISA assay as compared to the immunochromatographic test, but with an overall agreement of 91% (93/102) between the two tests. For the negative control sera, three of those produced contrasting results, from a total of 64, with a similar agreement overall (95%).

For the VL-positive canine sera, the two tests were also assessed with the same set of 198 VL-positive canine sera, including sera from BA, which were included in the previous analysis of the ELISA-Q5. Eleven were found to be negative for both tests, with only one test found to be negative with the ELISA-Q5, but positive for the RDT-Q5, while 17 sera, which were found negative for the rapid test, produced positive results using the ELISA-Q5 (~91% agreement overall). For the VL-negative canine sera, from a total of 47, an agreement of ~98% was observed between the two tests, with only one serum producing a false positive result for the RDT-Q5 only, and another producing a false positive result for both the ELISA-Q5 and RDT-Q5. This analysis confirms the substantial agreement between the two tests based on the Q5 protein.

## DISCUSSION

The current assessment of the chimeric Q5 expands on its previous evaluation as the basis of a novel ELISA assay with possible use for the VL diagnosis.^32^ The same assay was used more recently to evaluate sera from dogs diagnosed with VL based on commercial serological tests currently recommended by public health authorities, namely the EIE-LVC and DPP tests, but the results regarding the efficiency of the ELISA-Q5 were mainly inconclusive.^34^ Here, assessing sera from multiple localities and in greater numbers, we were able to confirm the versatility of using this PQ for the VL diagnosis in both humans and dogs, with a noticeably better performance for the canine sera.

This versatility is supported by the results using the RDT-Q5, first described in the current study, which was based on a setup with another antigen previously tested for VL diagnosis in dogs.^35^ Our results show a very efficient and consistent performance for the RDT-Q5 diagnosis regarding the human form of the disease, while showing its potential use also with the canine VL, but which would still need some improvement.

The need for new diagnostic tools that are rapid, cost-effective, and efficient, for the monitoring and treatment of human and canine VL is highlighted by the current dependency of public health authorities on tests still based on crude native proteins extracted from cultured parasites. Current assays are also lacking in efficiency for the diagnosis of immunocompromised individuals, such as those co-infected with human immunodeficiency virus (HIV), or impacted by age,^5^ ^,^ ^36^ ^,^ ^37^ ^,^ ^38^ and asymptomatic cases, mainly in dogs.^6^ ^,^ ^9^ Indeed, several recent publications still investigate or describe new antigens aiming at their potential use for serological VL diagnosis,^26^ ^,^ ^27^ ^,^ ^28^ ^,^ ^39^ ^-^ ^43^ confirming the need for further work aiming to improve current diagnostic methods.

Early studies considered using chimeric antigens to maximise their potential use for VL diagnosis using ELISA assays. An example is the PQ, derived from ribosomal proteins and histones, but with a lower than 80% sensitivity for the VL diagnosis in dogs.^44^ PQs based on the K9/K26/K39 antigens were later evaluated, and a greater performance (~95% sensitivity) was seen for the canine VL,^19^ with a related protein currently being the basis for the DPP test.^20^ A similar performance with canine VL was seen for the synthetic, multiepitope, PQ10^26^ ^,^ ^45^ and, as seen here for Q5, the recombinant PQ10 had a lower, but still relevant, efficiency with human VL sera (>80% sensitivity).^42^ An attempt for a better PQ to be used for the human VL diagnosis was the synthetic glucose-regulated protein 78 (GRP78), ubiquitin-conjugating enzyme E2, calreticulin, mitochondrial heat shock 70-related protein 1 (mtHSP70) (GRP-UBI-HSP), another multiepitope protein, but a limited sensitivity (~70%) was observed for the recombinant protein.^41^ The chimeric Q5 seems to be at least as efficient as the best PQ previously assessed for the diagnosis of both human and canine VL, reinforcing it as a viable alternative for the VL diagnosis, with potential use for both forms of the disease with for the diagnosis of human or canine VL. It contrasts with the performance of the individual proteins from which it was derived, either effective for the diagnosis of human or canine VL but not for both, and validates the approach used to improve its efficiency.^32^

The performance of the immunochromatographic RDT-Q5 with the human VL sera qualifies it as a cost-effective alternative for the diagnosis of human infection. Nevertheless, the comparative analysis between the ELISA and RDT assays indicates differences in sensitivity between the two tests regarding the canine and human sera, which are not directly related to the antigen used. Differences in sensitivity between ELISA and rapid test assays have also been reported for the synthetic rKDDR-plus antigen, derived from rK39, when both were evaluated for the human VL diagnosis, but with the ELISA having a better performance.^43^ These differences might be attributed to the use of a microtiter plate compared to a nitrocellulose membrane, secondary antibodies, or even blocking reagents. Alternatively, improvements on the recombinant antigen, possibly through the enhancement of its antigenic diversity, might further increase the efficiency of either or both tests and facilitate their use for both human and canine VL.

VL and HIV-acquired immunodeficiency syndrome (AIDS) coinfection is considered a life-threatening pathology when undiagnosed and untreated, due to the immunosuppression caused by both diseases.^37^ Commercially available tests currently used in Brazil for human VL diagnosis are based on rK39, which is not effective for immunocompromised individuals.^46^ The recombinant Lci2, one of the three proteins whose fragments were used to produce the chimeric Q5, is more effective for the VL diagnosis of VL/HIV co-infections,^47^ so a possible use of the RDT-Q5 in these situations can also be considered. Likewise, asymptomatic dogs are fully competent for VL transmission^48^ despite having lower antibody titres, leading to an impaired diagnosis by serological methods.^6^ ^,^ ^9^ ^,^ ^20^ ^,^ ^49^ The chimeric PQ10 antigen and the related PQ20 were specifically investigated regarding their potential for the diagnosis of naturally and experimentally infected dogs. Both were more efficient in identifying asymptomatic dogs infected with *L. infantum* than a serological assay with crude *Leishmania* antigens, with the results from the immunoassay relating to parasite load.^27^ ^,^ ^42^

The DPP® technology, used in the currently recommended diagnostic test for canine VL, is characterised by having different openings for loading of the sample (analyte) to be tested and for the buffer solution required for the diagnostic reaction to proceed. The analyte of interest migrates along the first membrane, binding to the capture agents immobilised in the test region. The buffer then allows the labelled ligands (probe) to migrate along a second membrane and bind to the analyte captured in the test region.^50^ In contrast, the Lateral Flow procedure used here for the RDT-Q5 consists of a single multimembrane strip arranged sequentially under an adhesive card. The strip is placed inside a plastic cassette where the reaction occurs. This device has a single opening for dispensing the sample/buffer, and its simpler design should be more cost-effective without loss of reliability.^33^ The performance of the RDT-Q5 with asymptomatic animals still needs to be evaluated, but based on the results seen so far, it might be considered a relevant alternative for their diagnosis in its current format or after further improvements.

The results shown here then confirm the potential for the chimeric Q5 as a promising new tool for the VL diagnosis of both human and canine forms of the disease. Its performance within the new RDT test, which is most likely to be used in general, validates its use as it is for the human VL diagnosis. Still, the recombinant Q5 can also be evaluated as part of a multiplex test (with several recombinant antigens). Further performance improvement can also be considered, especially for its use in canine VL diagnosis and for other animal species. As previously shown during the Q5 design,^32^ this may be achievable with reasonable ease and speed with the inclusion of new antigenic epitopes within its sequence, leading to an RDT equally effective for human and animal diagnosis to be used cost-effectively in the field as well as in laboratory settings.

Subsequent studies will require as much as possible the use of sera from individuals with the VL diagnosis confirmed parasitologically, or at least through PCR, to avoid bias introduced by previous testing with other serological methods, and which might partially be a reason for the reduced sensitivity observed here with the ELISA-Q5 for both human and canine samples from the State of PE. A larger number of negative human and canine samples also need to be included, as well as an evaluation of sera from individuals with other associated diseases, to assess cross-reactions. For the evaluation with the canine sera, these shall include sera from animals with confirmed diagnosis for diseases caused by other trypanosomatids, as well as *Ehrlichia canis*, *Babesia canis*, and others. Considering the human sera, a greater number of samples from individuals with CD and CL should be evaluated, as well, sera from individuals afflicted with other diseases. Further validation of the test will require its assessment with whole blood samples under simulated or real field conditions, in order to confirm the test’s applicability and its robustness in real-world settings. If the RDT-Q5 performance is confirmed effective for both human and canine VL, the standardised conditions allowing the same rapid test to be used for diagnosing both humans and dogs in field settings should greatly facilitate the streamlined production of a single test for both hosts and improve its cost-effectiveness and widespread use.

## SUPPLEMENTARY MATERIALS

Supplementary material

## Data Availability

The datasets generated and analysed during the current study are included in this published article and its supplementary material. Additional raw data supporting the findings of this study are available from the corresponding authors upon reasonable request. All sensitivity, specificity, and agreement calculations were based on the data provided in the Supplementary data (Table).
