# Supplementary material for: Evaluation of a new rapid diagnostic test, based on the chimeric protein Q5, for the diagnosis of human and canine forms of visceral leishmaniasis
Source: Mem Inst Oswaldo Cruz. 2026 May 11;121:e250126. doi: 10.1590/0074-02760250126 (PMC13182565; doi:10.1590/0074-02760250126)
Supplement: Supplementary material [file 1678-8060-mioc-121-e250126-s1.pdf]

| ID | Brazilian State | Parasitological | ELISA SLA | ELISA rK39 | RDT rK39 | IFI | PCR | HIV | DAT | ELISA Q5 | D.O | RDT Q5/ 2019 | RDT Q5/ 2017 | RDT Q5/ 2021 | RDT Lci2 |
|---|---|---|---|---|---|---|---|---|---|---|---|---|---|---|---|
| 3 | MS | POS | POS | POS | POS | POS | POS | NEG | POS | POS | 0,252 | POS | | POS | POS |
| 8 | MS | NEG | POS | POS | POS | POS | POS | NEG | POS | POS | 0,812 | POS | | POS | POS |
| 13 | MS | NEG | POS | POS | POS | POS | POS | NEG | | POS | 0,350 | POS | | POS | POS |
| 14 | MS | NEG | POS | POS | POS | POS | POS | NEG | NEG | POS | 0,433 | POS | | POS | POS |
| 15 | MS | POS | POS | POS | POS | POS | POS | NEG | POS | NEG | 0,027 | POS | | POS | POS |
| 19 | MS | POS | POS | POS | POS | POS | POS | NEG | | POS | 0,722 | POS | | POS | POS |
| 21 | MS | POS | POS | POS | POS | NEG | POS | NEG | POS | POS | 0,517 | POS | | POS | POS |
| 29 | MS | POS | POS | POS | POS | POS | POS | NEG | POS | POS | 0,713 | POS | | POS | POS |
| 30 | MS | NEG | POS | | NEG | POS | POS | NEG | | NEG | 0,017 | NEG | | POS | POS |
| 32 | MS | POS | POS | POS | POS | NEG | POS | NEG | POS | POS | 0,886 | POS | | POS | POS |
| 34 | MS | POS | POS | POS | POS | NEG | POS | NEG | POS | POS | 0,604 | POS | | POS | POS |
| 35 | MS | POS | POS | NEG | POS | NEG | POS | NEG | NEG | POS | 0,227 | POS | | POS | POS |
| 40 | MS | POS | POS | | POS | POS | POS | NEG | NEG | POS | 0,555 | POS | | POS | POS |
| 46 | MS | POS | POS | POS | POS | POS | POS | NEG | POS | POS | 0,346 | POS | | POS | POS |
| 48 | MS | POS | POS | POS | POS | POS | POS | NEG | POS | POS | 0,422 | POS | | POS | POS |
| 49 | MS | POS | POS | POS | POS | POS | POS | NEG | POS | POS | 0,565 | POS | | POS | POS |
| 52 | MS | POS | POS | POS | POS | POS | POS | NEG | POS | POS | 0,666 | POS | | POS | POS |
| 56 | MS | POS | POS | POS | POS | POS | POS | NEG | NEG | POS | 0,472 | POS | | POS | POS |
| 58 | MS | POS | POS | POS | POS | NEG | POS | NEG | POS | NEG | 0,025 | POS | | POS | POS |
| 64 | MS | POS | POS | POS | POS | NEG | POS | NEG | POS | POS | 0,395 | POS | | POS | POS |
| 65 | MS | POS | POS | POS | POS | POS | POS | NEG | POS | POS | 0,274 | POS | | POS | NEG |
| 71 | MS | POS | POS | POS | POS | POS | POS | NEG | POS | POS | 0,364 | POS | | POS | POS |
| 74 | MS | POS | POS | POS | | NEG | POS | NEG | POS | POS | 0,251 | POS | | POS | POS |
| 75 | MS | NEG | POS | NEG | POS | POS | POS | NEG | POS | POS | 0,199 | POS | | POS | POS |
| 77 | MS | POS | POS | POS | POS | NEG | POS | NEG | POS | POS | 0,524 | POS | | | |
| 79 | MS | POS | POS | POS | POS | POS | POS | NEG | POS | POS | 0,621 | POS | | | |
| 85 | MS | NEG | POS | POS | POS | POS | POS | NEG | POS | POS | 0,181 | POS | | | |
| 86 | MS | POS | NEG | POS | POS | POS | POS | NEG | POS | POS | 0,249 | POS | | | |
| 91 | MS | POS | POS | NEG | POS | POS | POS | NEG | POS | POS | 0,517 | POS | | | |
| 94 | MS | POS | POS | POS | | POS | POS | NEG | POS | POS | 0,248 | POS | | | |
| 2885 | PI | POS | POS | NEG | NEG | POS | | NEG | POS | NEG | 0,005 | NEG | | | |
| 2886 | PI | POS | POS | POS | POS | POS | | NEG | POS | POS | 0,158 | POS | POS | | |
| 2921 | PI | POS | POS | POS | POS | POS | | NEG | POS | POS | 2,869 | POS | POS | | |
| 2924 | PI | POS | POS | POS | POS | POS | | NEG | POS | POS | 1,039 | POS | POS | | |
| 2936 | PI | POS | POS | POS | POS | POS | | NEG | POS | POS | 0,390 | POS | POS | POS | POS |
| 2969 | PI | POS | NEG | NEG | NEG | POS | | NEG | NEG | NEG | 0,059 | NEG | NEG | | |
| 2974 | PI | POS | POS | POS | POS | POS | | NEG | POS | POS | 1,140 | POS | POS | POS | POS |
| 2975 | PI | POS | POS | POS | POS | POS | | NEG | POS | POS | 0,791 | POS | POS | POS | POS |
| 2976 | PI | POS | POS | POS | POS | POS | | NEG | POS | POS | 2,093 | POS | POS | POS | POS |
| 2978 | PI | POS | POS | POS | NEG | POS | | NEG | POS | POS | 0,245 | POS | POS | POS | POS |
| 2981 | PI | POS | POS | POS | POS | POS | | NEG | POS | POS | 1,554 | POS | POS | POS | POS |
| 2982 | PI | POS | POS | POS | POS | POS | | NEG | POS | POS | 1,606 | POS | POS | POS | POS |
| 3002 | PI | POS | POS | NEG | POS | NEG | | NEG | POS | NEG | 0,026 | POS | POS | POS | NEG |
| 3003 | PI | POS | POS | POS | POS | POS | | NEG | POS | POS | 0,286 | POS | POS | POS | POS |
| 3021 | PI | POS | POS | POS | POS | POS | | NEG | POS | POS | 2,386 | POS | POS | POS | POS |
| 3025 | PI | POS | POS | NEG | POS | NEG | | NEG | POS | POS | 1,415 | POS | POS | POS | POS |

| ID | Brazilian State | Parasitological | ELISA SLA | ELISA rK39 | RDT rK39 | IFI | PCR | HIV | DAT | ELISA Q5 | D.O | RDT Q5/ 2019 | RDT Q5/ 2017 | RDT Q5/ 2021 | RDT Lci2 |
|---|---|---|---|---|---|---|---|---|---|---|---|---|---|---|---|
| 3027 | PI | POS | POS | POS | POS | POS | | NEG | POS | NEG | 0,006 | POS | POS | POS | POS |
| 3031 | PI | POS | POS | POS | POS | NEG | | NEG | POS | POS | 0,298 | POS | POS | NEG | POS |
| 3038 | PI | POS | POS | POS | POS | POS | | NEG | POS | POS | 1,553 | POS | POS | POS | POS |
| 3039 | PI | POS | POS | POS | POS | POS | | NEG | POS | POS | 2,237 | POS | POS | POS | POS |
| 3045 | PI | POS | POS | POS | POS | POS | | NEG | POS | POS | 0,243 | POS | POS | POS | POS |
| 3049 | PI | POS | POS | POS | POS | NEG | | NEG | POS | POS | 0,689 | POS | POS | POS | POS |
| 3050 | PI | POS | POS | POS | POS | NEG | | NEG | POS | POS | 0,312 | POS | POS | POS | POS |
| 3060 | PI | POS | POS | POS | POS | POS | | NEG | POS | POS | 1,453 | POS | POS | | |
| 3075 | PI | POS | POS | POS | POS | POS | | NEG | POS | POS | 1,302 | POS | POS | | |
| 3080 | PI | POS | POS | NEG | POS | POS | | NEG | POS | POS | 0,243 | POS | POS | | |
| 3081 | PI | POS | POS | POS | POS | NEG | | NEG | POS | POS | 2,152 | POS | POS | | |
| 3087 | PI | POS | POS | POS | POS | NEG | | NEG | POS | POS | 0,325 | POS | POS | | |
| 3113 | PI | POS | POS | POS | POS | POS | | NEG | POS | POS | 0,933 | POS | POS | | |
| 3115 | PI | POS | POS | POS | POS | POS | | NEG | POS | POS | 1,311 | POS | POS | | |
| 3116 | PI | POS | POS | POS | POS | POS | | NEG | POS | POS | 1,340 | POS | POS | | |
| 3120 | PI | POS | POS | POS | POS | POS | | NEG | POS | POS | 2,134 | POS | POS | | |
| 3121 | PI | POS | POS | POS | POS | POS | | NEG | POS | POS | 0,988 | POS | POS | POS | POS |
| 3126 | PI | POS | POS | POS | POS | POS | | NEG | POS | POS | 2,151 | POS | POS | | |
| 3127 | PI | POS | POS | POS | POS | POS | | NEG | POS | NEG | 0,012 | POS | POS | | |
| 3130 | PI | POS | POS | POS | POS | POS | | NEG | POS | POS | 1,337 | POS | POS | POS | POS |
| 3132 | PI | POS | POS | POS | POS | POS | | NEG | POS | POS | 0,262 | POS | POS | | |
| 3133 | PI | POS | POS | NEG | NEG | NEG | | NEG | POS | NEG | 0,018 | NEG | NEG | NEG | POS |
| 3134 | PI | POS | POS | POS | POS | POS | | NEG | POS | POS | 0,234 | POS | POS | | |
| 3142 | PI | POS | POS | POS | POS | POS | | NEG | POS | POS | 1,550 | POS | POS | | |
| 3151 | PI | POS | POS | POS | POS | POS | | NEG | POS | POS | 1,079 | POS | POS | | |
| 3908 | PE | POS | POS | POS | POS | NEG | | NEG | POS | POS | 0,270 | POS | | | |
| 4162 | PE | POS | POS | POS | POS | NEG | | NEG | POS | NEG | 0,008 | NEG | | | |
| 3101 | PE | POS | POS | POS | POS | POS | | NEG | POS | POS | 0,272 | POS | | | |
| 3946 | PE | POS | POS | POS | POS | POS | POS | NEG | POS | POS | 0,352 | POS | | | |
| 3973 | PE | POS | POS | POS | POS | POS | POS | NEG | POS | POS | 0,345 | POS | | | |
| 4099 | PE | POS | POS | POS | POS | POS | POS | NEG | POS | POS | 0,236 | POS | | | |
| 4112 | PE | POS | POS | POS | POS | POS | | NEG | POS | POS | 0,316 | POS | | | |
| CAZ 1022 | PE | POS | POS | | POS | POS | | NEG | POS | POS | 0,238 | POS | | | |
| CAZ 992 | PE | POS | POS | | NEG | POS | POS | NEG | POS | POS | 0,327 | POS | | | |
| 3929 | PE | | NEG | NEG | POS | NEG | POS | NEG | POS | NEG | 0,008 | POS | | | |
| 4114 | PE | | POS | POS | | POS | | NEG | POS | POS | 0,486 | POS | | | |
| 4166 | PE | | POS | POS | POS | POS | POS | NEG | POS | POS | 0,192 | POS | | | |
| 4144 | PE | | POS | POS | POS | POS | POS | NEG | POS | POS | 0,327 | POS | | | |
| 4137 | PE | | POS | POS | POS | POS | POS | NEG | POS | POS | 0,196 | POS | | | |
| 4845 | PE | | POS | POS | POS | POS | POS | NEG | | POS | 0,246 | POS | | | |
| CAZ 951 | PE | | POS | POS | POS | POS | POS | NEG | POS | POS | 0,253 | POS | | | |
| 4867 | PE | | POS | POS | POS | POS | POS | NEG | POS | POS | 0,353 | POS | | | |
| 4456 | PE | | POS | POS | POS | POS | POS | NEG | POS | POS | 0,172 | POS | | | |
| CAZ 963 | PE | | POS | | NEG | POS | NEG | NEG | POS | POS | 0,233 | POS | | | |
| 4229 | PE | | POS | | POS | POS | POS | NEG | POS | POS | 0,159 | POS | | | |
| 4873 | PE | | POS | | POS | POS | NEG | NEG | POS | POS | 0,235 | POS | | | |

| ID | Brazilian State | Parasitological | ELISA SLA | ELISA rK39 | RDT rK39 | IFI | PCR | HIV | DAT | ELISA Q5 | D.O | RDT Q5/ 2019 | RDT Q5/ 2017 | RDT Q5/ 2021 | RDT Lci2 |
|---|---|---|---|---|---|---|---|---|---|---|---|---|---|---|---|
| 4865 | PE | | POS | | POS | POS | NEG | NEG | POS | POS | 0,457 | POS | | | |
| 4300 | PE | | POS | | POS | POS | POS | NEG | POS | POS | 0,246 | POS | | | |
| CAZ 980 | PE | | POS | | POS | POS | POS | NEG | POS | NEG | 0,006 | POS | | | |
| CAZ 995 | PE | | POS | | POS | POS | | NEG | POS | NEG | 0,017 | NEG | | | |
| CAZ 989 | PE | | POS | | POS | POS | NEG | NEG | POS | POS | 0,389 | POS | | | |
| CAZ 990 | PE | | POS | | POS | POS | POS | NEG | POS | NEG | 0,018 | POS | | | |
| CAZ 977 | PE | | POS | | POS | POS | | NEG | POS | POS | 0,242 | POS | | | |
| CAZ 976 | PE | | POS | | POS | POS | POS | NEG | POS | NEG | 0,006 | POS | | | |
| CAZ 1033 | PE | | POS | | POS | POS | | NEG | POS | POS | 0,375 | POS | | | |
| CAZ 1035 | PE | | POS | | POS | POS | POS | NEG | POS | POS | 0,439 | POS | | | |

| ID | Brazilian State | Parasitological | ELISA SLA | ELISA rK39 | RDT rK39 | ELISA Q5 | D.O | RDT Q5/2019 | RDT Q5/2017 | RDT Q5/2021 | RDT - Lci2 | DAT | | |
|---|---|---|---|---|---|---|---|---|---|---|---|---|---|---|
| 1 | PE | NEG | NEG | NEG | NEG | NEG | 0,073 | NEG | | NEG | POS | NEG | | |
| 2 | PE | NEG | NEG | NEG | NEG | NEG | 0,034 | NEG | | NEG | NEG | NEG | MN | 0,035 |
| 3 | PE | NEG | NEG | NEG | NEG | NEG | 0,056 | NEG | | NEG | NEG | NEG | DP | 0,027 |
| 4 | PE | NEG | NEG | NEG | NEG | NEG | 0,024 | NEG | | NEG | POS | NEG | Cut-off | **0,089** |
| 5 | PE | NEG | NEG | NEG | NEG | NEG | 0,069 | NEG | | NEG | NEG | POS | | |
| 6 | PE | NEG | NEG | NEG | NEG | NEG | 0,057 | NEG | | NEG | NEG | NEG | | |
| 7 | PE | NEG | NEG | NEG | NEG | NEG | 0,062 | NEG | | NEG | NEG | NEG | | |
| 8 | PE | NEG | NEG | NEG | NEG | NEG | 0,086 | NEG | | NEG | NEG | NEG | | |
| 9 | PE | NEG | NEG | NEG | NEG | NEG | 0,050 | NEG | | NEG | NEG | NEG | | |
| 10 | PE | NEG | NEG | NEG | NEG | NEG | 0,056 | NEG | | NEG | NEG | NEG | | |
| 11 | PE | NEG | NEG | NEG | NEG | NEG | 0,061 | NEG | | NEG | NEG | NEG | | |
| 12 | PE | NEG | NEG | NEG | NEG | NEG | 0,072 | NEG | | NEG | NEG | NEG | | |
| 13 | PE | NEG | NEG | NEG | NEG | NEG | 0,073 | NEG | | NEG | NEG | NEG | | |
| 14 | PE | NEG | NEG | NEG | NEG | NEG | 0,062 | NEG | | NEG | NEG | NEG | | |
| 15 | PE | NEG | NEG | NEG | NEG | NEG | 0,059 | NEG | | NEG | POS | NEG | | |
| 16 | PE | NEG | NEG | POS | NEG | NEG | 0,078 | NEG | | NEG | NEG | NEG | | |
| 17 | PE | NEG | NEG | NEG | NEG | NEG | 0,074 | NEG | | NEG | NEG | NEG | | |
| 18 | PE | NEG | NEG | NEG | NEG | NEG | 0,064 | NEG | | NEG | NEG | NEG | | |
| 19 | PE | NEG | NEG | NEG | NEG | NEG | 0,081 | NEG | | NEG | NEG | NEG | | |
| 20 | PE | NEG | NEG | NEG | NEG | POS | **0,100** | NEG | | NEG | NEG | NEG | | |
| 21 | PE | NEG | NEG | NEG | NEG | NEG | 0,006 | NEG | | NEG | NEG | NEG | | |
| 22 | PE | NEG | NEG | NEG | NEG | NEG | 0,060 | NEG | | NEG | NEG | NEG | | |
| 23 | PE | NEG | NEG | NEG | NEG | NEG | 0,057 | NEG | | NEG | NEG | NEG | | |
| 24 | PE | NEG | NEG | NEG | NEG | NEG | 0,066 | NEG | | NEG | NEG | NEG | | |
| 25 | PE | NEG | NEG | NEG | NEG | NEG | 0,070 | NEG | | NEG | NEG | NEG | | |
| 26 | PE | NEG | NEG | NEG | NEG | NEG | 0,071 | NEG | | NEG | NEG | NEG | | |
| 28 | PE | NEG | NEG | NEG | NEG | NEG | 0,015 | NEG | | NEG | NEG | NEG | | |
| 29 | PE | NEG | NEG | NEG | NEG | NEG | 0,007 | NEG | | NEG | NEG | NEG | | |
| 30 | PE | NEG | NEG | NEG | NEG | NEG | 0,014 | NEG | | NEG | NEG | NEG | | |
| 31 | PE | NEG | NEG | NEG | NEG | NEG | 0,026 | NEG | | NEG | NEG | NEG | | |
| 32 | PE | NEG | NEG | NEG | NEG | NEG | 0,016 | NEG | | NEG | NEG | NEG | | |

| ID | Brazilian State | Parasitological | ELISA SLA | ELISA rK39 | RDT rK39 | ELISA Q5 | D.O | RDT Q5/2019 | RDT Q5/2017 | RDT Q5/2021 | RDT - Lci2 | DAT |
|---|---|---|---|---|---|---|---|---|---|---|---|---|
| 33 | PE | NEG | NEG | NEG | NEG | NEG | 0,020 | NEG | | NEG | NEG | NEG |
| 34 | PE | NEG | NEG | NEG | NEG | NEG | 0,032 | NEG | | NEG | NEG | NEG |
| 35 | PE | NEG | NEG | NEG | NEG | NEG | 0,004 | NEG | | NEG | NEG | NEG |
| 36 | PE | NEG | NEG | NEG | NEG | NEG | 0,019 | NEG | | NEG | NEG | NEG |
| 37 | PE | NEG | NEG | NEG | NEG | NEG | 0,013 | NEG | | NEG | NEG | NEG |
| 38 | PE | NEG | NEG | NEG | NEG | NEG | 0,005 | NEG | | NEG | NEG | NEG |
| 39 | PE | NEG | NEG | NEG | NEG | NEG | 0,013 | NEG | | NEG | NEG | NEG |
| 40 | PE | NEG | NEG | NEG | NEG | NEG | 0,001 | NEG | | NEG | NEG | NEG |
| 41 | PE | NEG | NEG | NEG | NEG | NEG | 0,012 | NEG | | NEG | NEG | NEG |
| 42 | PE | NEG | NEG | NEG | NEG | NEG | 0,001 | NEG | | NEG | | NEG |
| 43 | PE | NEG | NEG | NEG | NEG | NEG | 0,010 | NEG | | NEG | | NEG |
| 44 | PE | NEG | NEG | NEG | NEG | NEG | 0,008 | NEG | | NEG | | NEG |
| 45 | PE | NEG | NEG | NEG | NEG | NEG | 0,012 | NEG | | NEG | | NEG |
| 46 | PE | NEG | NEG | NEG | NEG | NEG | 0,018 | NEG | | NEG | | NEG |
| 47 | PE | NEG | NEG | NEG | NEG | NEG | 0,028 | NEG | | | | NEG |
| 48 | PE | NEG | NEG | NEG | NEG | NEG | 0,023 | NEG | | | | NEG |
| 49 | PE | NEG | NEG | NEG | NEG | NEG | 0,006 | NEG | | | | NEG |
| 50 | PE | NEG | NEG | NEG | NEG | NEG | 0,007 | NEG | | | | NEG |
| 3 | PE | NEG | NEG | NEG | NEG | NEG | 0,025 | NEG | NEG | | | |
| 4 | PE | NEG | NEG | NEG | NEG | NEG | 0,012 | NEG | NEG | | | |
| 6 | PE | NEG | NEG | NEG | NEG | NEG | 0,011 | POS | POS | | | |
| 13 | PE | NEG | NEG | NEG | NEG | NEG | 0,011 | NEG | NEG | | | |
| 14 | PE | NEG | NEG | NEG | NEG | NEG | 0,005 | NEG | NEG | | | |
| 15 | PE | NEG | NEG | NEG | NEG | NEG | 0,040 | POS | POS | | | |
| 16 | PE | NEG | NEG | NEG | NEG | NEG | 0,034 | NEG | NEG | | | |
| 17 | PE | NEG | NEG | NEG | NEG | NEG | 0,018 | NEG | NEG | | | |
| 23 | PE | NEG | NEG | NEG | NEG | NEG | 0,015 | NEG | NEG | | | |
| 27 | PE | NEG | NEG | NEG | NEG | NEG | 0,004 | NEG | NEG | | | |
| 32 | PE | NEG | NEG | NEG | NEG | NEG | 0,017 | NEG | | | | |
| 35 | PE | NEG | NEG | NEG | NEG | NEG | 0,029 | NEG | | | | |
| 37 | PE | NEG | NEG | NEG | NEG | NEG | 0,048 | NEG | | | | |
| 38 | PE | NEG | NEG | NEG | NEG | NEG | 0,037 | NEG | | | | |
| 44 | PE | NEG | NEG | NEG | NEG | NEG | 0,017 | NEG | | | | |

| ID | IDRM | ELISA Q5 | D.O | RDT Q5/2019 | RDT - Q5/2021 | RDT - Lci2 |
|---|---|---|---|---|---|---|
| 1 | POS | NEG | 0,020 | NEG | NEG | NEG |
| 2 | POS | NEG | 0,019 | NEG | NEG | NEG |
| 3 | POS | NEG | 0,015 | NEG | NEG | POS |
| 4 | POS | NEG | 0,022 | NEG | NEG | NEG |
| 5 | POS | POS | 0,089 | POS | NEG | POS |
| 6 | POS | NEG | 0,006 | NEG | NEG | POS |
| 7 | POS | NEG | 0,007 | NEG | NEG | POS |
| 8 | POS | NEG | 0,016 | NEG | NEG | POS |
| 9 | POS | POS | 0,113 | POS | NEG | POS |
| 10 | POS | NEG | 0,015 | NEG | NEG | NEG |
| 11 | POS | NEG | 0,031 | NEG | | |

| ID | IDRM | ELISA Q5 | D.O | RDT Q5/2019 | RDT - Q5/2021 | RDT - Lci2 |
|---|---|---|---|---|---|---|
| 12 | POS | NEG | 0,012 | NEG | | |
| 13 | POS | NEG | 0,016 | NEG | | |
| 14 | POS | NEG | 0,005 | NEG | | |
| 15 | POS | NEG | 0,017 | NEG | | |
| 16 | POS | NEG | 0,041 | NEG | | |
| 17 | POS | NEG | 0,026 | NEG | | |
| 18 | POS | NEG | 0,028 | NEG | | |
| 19 | POS | NEG | 0,023 | NEG | | |
| 20 | POS | NEG | 0,018 | NEG | | |
| 21 | POS | NEG | 0,007 | NEG | | |
| 22 | POS | NEG | 0,017 | NEG | | |
| 23 | POS | NEG | 0,057 | NEG | | |
| 24 | POS | NEG | 0,008 | NEG | | |
| 25 | POS | NEG | 0,019 | NEG | | |

| ID | Elisa Q5 | D.O | RDT Q5/2019 | RDT - Q5/2021 | RDT - Lci2 |
|---|---|---|---|---|---|
| 1 | NEG | 0,052 | NEG | NEG | NEG |
| 2 | NEG | 0,077 | NEG | NEG | POS |
| 10 | NEG | 0,065 | NEG | NEG | NEG |
| 11 | NEG | 0,062 | NEG | NEG | POS |
| 12 | NEG | 0,072 | NEG | NEG | NEG |
| 20 | NEG | 0,088 | NEG | NEG | NEG |
| 21 | NEG | 0,071 | NEG | NEG | NEG |
| 26 | NEG | 0,063 | NEG | NEG | POS |
| 28 | NEG | 0,066 | NEG | NEG | POS |
| 30 | NEG | 0,087 | NEG | NEG | NEG |

| ID | Brazilian State | Parasitological | ELISA SLA | ELISA rK39 | RDT rK39 | IFI | PCR | ELISA Q5 | D.O | DPP - LVC | RDT Q5/2019 | RDT Q5/2017 | RDT Q5/2021 |
|---|---|---|---|---|---|---|---|---|---|---|---|---|---|
| 391 | MS | POS | POS | | | | | POS | 1,863 | POS | POS | | POS |
| 419 | MS | POS | POS | | | | | POS | 1,696 | POS | POS | | POS |
| 90 | MS | POS | POS | | | | | POS | 1,632 | POS | POS | | POS |
| 222 | MS | POS | POS | | | | | POS | 1,846 | POS | POS | | POS |
| 322 | MS | POS | POS | | | | | POS | 0,990 | POS | NEG | | NEG |
| 88 | MS | POS | POS | | | | | POS | 1,713 | POS | POS | | POS |
| 274 | MS | POS | POS | | | | | POS | 1,754 | POS | POS | | POS |
| 373 | MS | POS | POS | | | | | POS | 1,807 | POS | POS | | POS |
| 191 | MS | POS | POS | | | | | POS | 1,869 | POS | POS | | POS |
| 294 | MS | POS | POS | | | | | POS | 1,870 | POS | POS | | POS |
| 266 | MS | POS | POS | | | | | POS | 1,379 | POS | NEG | | POS |
| 408 | MS | POS | POS | | | | | POS | 1,612 | POS | POS | | POS |
| 300 | MS | POS | POS | | | | | POS | 1,849 | POS | POS | | POS |
| 78 | MS | POS | POS | | | | | POS | 1,607 | POS | POS | | POS |
| 235 | MS | POS | POS | | | | | POS | 1,721 | POS | POS | | POS |
| 340 | MS | POS | POS | | | | | POS | 1,690 | POS | POS | | POS |
| 239 | MS | POS | POS | | | | | POS | 1,908 | POS | POS | | POS |

| ID | Brazilian State | Parasitological | ELISA SLA | ELISA rK39 | RDT rK39 | IFI | PCR | ELISA Q5 | D.O | DPP - LVC | RDT Q5/2019 | RDT Q5/2017 | RDT Q5/2021 |
|---|---|---|---|---|---|---|---|---|---|---|---|---|---|
| 267 | MS | POS | POS | | | | | POS | 1,877 | POS | POS | | POS |
| 142 | MS | POS | POS | | | | | POS | 1,383 | POS | POS | | POS |
| 390 | MS | POS | POS | | | | | POS | 1,642 | POS | POS | | POS |
| 152 | MS | POS | POS | | | | | POS | 1,838 | POS | POS | | POS |
| 248 | MS | POS | POS | | | | | POS | 1,391 | POS | POS | | POS |
| 281 | MS | POS | POS | | | | | POS | 1,873 | POS | POS | | POS |
| 280 | MS | POS | POS | | | | | POS | 0,749 | POS | NEG | | NEG |
| 406 | MS | POS | POS | | | | | POS | 1,951 | POS | POS | | POS |
| 277 | MS | POS | POS | | | | | POS | 1,600 | POS | POS | | POS |
| 339 | MS | POS | POS | | | | | POS | 0,910 | POS | POS | | POS |
| 219 | MS | POS | POS | | | | | POS | 1,567 | POS | POS | | POS |
| 384 | MS | POS | POS | | | | | POS | 1,787 | POS | POS | | POS |
| 198 | MS | POS | POS | | | | | POS | 1,600 | POS | POS | | POS |
| 297 | MS | POS | POS | | | | | POS | 1,765 | POS | POS | | POS |
| 250 | MS | POS | POS | | | | | POS | 1,716 | POS | POS | | POS |
| 290 | MS | POS | POS | | | | | POS | 1,460 | POS | POS | | POS |
| 335 | MS | POS | POS | | | | | POS | 1,565 | POS | POS | | POS |
| 293 | MS | POS | POS | | | | | POS | 0,493 | POS | NEG | | NEG |
| 127 | MS | POS | POS | | | | | POS | 1,540 | POS | POS | | POS |
| 275 | MS | POS | POS | | | | | POS | 1,740 | POS | POS | | POS |
| 411 | MS | POS | POS | | | | | POS | 1,280 | POS | POS | | POS |
| 79 | MS | POS | POS | | | | | POS | 1,434 | POS | POS | | POS |
| 325 | MS | POS | POS | | | | | POS | 1,589 | POS | POS | | POS |
| 341 | MS | POS | POS | | | | | POS | 1,726 | POS | POS | | POS |
| 338 | MS | POS | POS | | | | | POS | 1,617 | POS | POS | | POS |
| 401 | MS | POS | POS | | | | | POS | 1,691 | POS | POS | | POS |
| 295 | MS | POS | POS | | | | | POS | 1,510 | POS | POS | | POS |
| 382 | MS | POS | POS | | | | | POS | 1,351 | POS | POS | | POS |
| 324 | MS | POS | POS | | | | | POS | 1,506 | POS | POS | | POS |
| 188 | MS | POS | POS | | | | | POS | 1,724 | POS | POS | | |
| 364 | MS | POS | POS | | | | | POS | 1,507 | POS | POS | | |
| 271 | MS | POS | POS | | | | | POS | 1,809 | POS | POS | | |
| 337 | MS | POS | POS | | | | | POS | 1,663 | POS | POS | | |
| 227 | MS | POS | POS | | | | | POS | 1,866 | POS | POS | | |
| 342 | MS | POS | POS | | | | | POS | 1,683 | POS | POS | | |
| 405 | MS | POS | POS | | | | | POS | 1,562 | POS | POS | | |
| 301 | MS | POS | POS | | | | | POS | 1,231 | POS | POS | | |
| 131 | MS | POS | POS | | | | | POS | 0,785 | POS | NEG | | |
| 319 | MS | POS | POS | | | | | POS | 1,156 | POS | POS | | |
| 396 | MS | POS | POS | | | | | POS | 0,320 | POS | NEG | | |
| 95 | MS | POS | POS | | | | | POS | 1,517 | POS | POS | | |
| 336 | MS | POS | POS | | | | | POS | 1,306 | POS | POS | | |
| 257 | MS | POS | POS | | | | | POS | 1,808 | POS | POS | | |
| 402 | MS | POS | POS | | | | | POS | 1,049 | POS | POS | | |
| 385 | MS | POS | POS | | | | | POS | 1,875 | POS | POS | | |
| 378 | MS | POS | POS | | | | | POS | 1,860 | POS | POS | | |
| 85 | MS | POS | POS | | | | | POS | 1,744 | POS | POS | | |

| ID | Brazilian State | Parasitological | ELISA SLA | ELISA rK39 | RDT rK39 | IFI | PCR | ELISA Q5 | D.O | DPP - LVC | RDT Q5/2019 | RDT Q5/2017 | RDT Q5/2021 |
|---|---|---|---|---|---|---|---|---|---|---|---|---|---|
| 334 | MS | POS | POS | | | | | POS | 0,532 | POS | POS | | |
| 412 | MS | POS | POS | | | | | NEG | 0,037 | POS | NEG | | |
| 279 | MS | POS | POS | | | | | POS | 1,603 | POS | POS | | |
| 323 | MS | POS | POS | | | | | POS | 1,511 | POS | POS | | |
| 389 | MS | POS | POS | | | | | POS | 1,565 | POS | POS | | |
| 331 | MS | POS | POS | | | | | POS | 1,853 | POS | POS | | |
| 111 | MS | POS | POS | | | | | POS | 0,450 | POS | POS | | |
| 318 | MS | POS | POS | | | | | POS | 1,837 | POS | POS | | |
| 217 | MS | POS | POS | | | | | POS | 1,854 | POS | POS | | |
| 285 | MS | POS | POS | | | | | POS | 1,735 | POS | POS | | |
| 362 | MS | POS | POS | | | | | POS | 1,701 | POS | POS | | |
| 263 | MS | POS | POS | | | | | POS | 1,336 | POS | POS | | |
| 357 | MS | POS | POS | | | | | POS | 1,923 | POS | POS | | |
| 253 | MS | POS | POS | | | | | POS | 1,790 | POS | POS | | |
| 121 | MS | POS | POS | | | | | POS | 1,575 | POS | POS | | |
| 284 | MS | POS | POS | | | | | POS | 1,572 | NEG | POS | | |
| 273 | MS | POS | POS | | | | | POS | 3,393 | POS | POS | | |
| 387 | MS | POS | POS | | | | | POS | 2,687 | POS | POS | | |
| 272 | MS | POS | POS | | | | | POS | 3,027 | POS | POS | | |
| 302 | MS | POS | POS | | | | | POS | 2,094 | POS | POS | | |
| 286 | MS | POS | POS | | | | | POS | 2,420 | POS | POS | | |
| 289 | MS | POS | POS | | | | | POS | 3,381 | POS | POS | | |
| 216 | MS | POS | POS | | | | | POS | 1,133 | POS | POS | | |
| 397 | MS | POS | POS | | | | | POS | 1,130 | POS | POS | | |
| 147 | MS | POS | POS | | | | | POS | 2,962 | POS | POS | | |
| 278 | MS | POS | POS | | | | | POS | 3,182 | POS | POS | | |
| 241 | MS | POS | POS | | | | | POS | 2,417 | POS | POS | | |
| 333 | MS | POS | POS | | | | | POS | 2,827 | POS | POS | | |
| 98 | MS | POS | POS | | | | | POS | 3,490 | POS | POS | | |
| 327 | MS | POS | POS | | | | | POS | 1,389 | POS | POS | | |
| 360 | MS | POS | POS | | | | | POS | 1,800 | POS | POS | | |
| 395 | MS | POS | POS | | | | | POS | 2,844 | POS | POS | | |
| 298 | MS | POS | POS | | | | | POS | 0,888 | POS | POS | | |
| 394 | MS | POS | POS | | | | | POS | 2,067 | POS | POS | | |
| 203 | MS | POS | POS | | | | | POS | 0,404 | POS | NEG | | |
| 238 | MS | POS | POS | | | | | POS | 2,877 | POS | POS | | |
| 272 | BA | POS | POS | | | | POS | POS | 2,467 | POS | POS | POS | POS |
| 300 | BA | POS | POS | POS | | | NEG | NEG | 0,095 | NEG | NEG | NEG | NEG |
| 263 | BA | POS | POS | POS | | | POS | POS | 1,399 | POS | POS | POS | POS |
| 324 | BA | POS | POS | | | | | POS | 2,228 | POS | POS | POS | POS |
| 322 | BA | POS | POS | POS | | | | POS | 1,091 | POS | NEG | POS | NEG |
| 256 | BA | POS | | | | | POS | POS | 1,365 | POS | POS | POS | POS |
| 313 | BA | POS | POS | POS | | | | POS | 2,401 | POS | NEG | POS | NEG |
| 316 | BA | POS | POS | POS | | | | POS | 2,161 | NEG | NEG | POS | NEG |
| 337 | BA | POS | POS | POS | | | | POS | 1,020 | POS | NEG | POS | NEG |
| 361 | BA | POS | POS | | | | | POS | 1,159 | POS | POS | POS | POS |
| 330 | BA | POS | | | | | | POS | 2,037 | POS | POS | POS | POS |

| ID | Brazilian State | Parasitological | ELISA SLA | ELISA rK39 | RDT rK39 | IFI | PCR | ELISA Q5 | D.O | DPP - LVC | RDT Q5/2019 | RDT Q5/2017 | RDT Q5/2021 |
|---|---|---|---|---|---|---|---|---|---|---|---|---|---|
| 307 | BA | POS | POS | | | | | POS | 1,267 | POS | POS | POS | POS |
| 315 | BA | POS | NEG | | | | | POS | 0,822 | POS | POS | POS | POS |
| 323 | BA | POS | POS | | | | | NEG | 0,206 | NEG | NEG | POS | NEG |
| 366 | BA | POS | POS | | | | | POS | 1,492 | POS | POS | POS | POS |
| 266 | BA | POS | POS | | | | POS | NEG | 0,209 | POS | NEG | POS | POS |
| 265 | BA | POS | POS | | | | POS | POS | 1,738 | POS | POS | POS | POS |
| 301 | BA | POS | POS | POS | | | POS | POS | 1,967 | POS | POS | POS | POS |
| 318 | BA | POS | POS | | | | | POS | 1,694 | POS | POS | POS | POS |
| 283 | BA | POS | POS | POS | | | POS | POS | 0,291 | POS | NEG | POS | POS |
| 331 | BA | POS | POS | POS | | | | POS | 2,232 | POS | NEG | POS | NEG |
| 358 | BA | POS | POS | | | | | POS | 1,400 | POS | POS | POS | POS |
| 348 | BA | POS | POS | | | | | POS | 0,638 | POS | POS | POS | POS |
| 352 | BA | POS | POS | POS | | | | POS | 0,864 | POS | POS | POS | POS |
| 362 | BA | POS | POS | | | | | POS | 2,327 | POS | POS | POS | POS |
| 275 | BA | POS | POS | | | | POS | POS | 1,205 | POS | POS | POS | POS |
| 365 | BA | POS | POS | | | | | POS | 1,399 | POS | POS | POS | POS |
| 367 | BA | POS | POS | | | | | POS | 2,148 | POS | NEG | POS | POS |
| 305 | BA | POS | POS | | | | | POS | 0,681 | POS | POS | POS | POS |
| 274 | BA | POS | POS | | | | POS | POS | 2,270 | POS | POS | POS | POS |
| 329 | BA | POS | POS | | | | | POS | 1,467 | POS | POS | POS | POS |
| 291 | BA | POS | POS | POS | | | POS | NEG | 0,138 | POS | NEG | POS | NEG |
| 295 | BA | POS | POS | POS | | | POS | POS | 1,997 | POS | POS | POS | POS |
| 290 | BA | POS | POS | | | | POS | POS | 1,236 | POS | POS | POS | POS |
| 327 | BA | POS | POS | | | | | POS | 2,210 | POS | POS | POS | POS |
| 260 | BA | POS | POS | POS | | | POS | NEG | 0,160 | POS | NEG | POS | NEG |
| 306 | BA | POS | POS | | | | | POS | 2,129 | POS | POS | POS | |
| 2 | PE | | POS | | | POS | POS | POS | 0,911 | POS | POS | | |
| 4 | PE | | POS | | | POS | NEG | POS | 0,588 | POS | POS | | |
| 12 | PE | | POS | | | POS | NEG | POS | 0,446 | POS | POS | | |
| 13 | PE | | POS | | | POS | POS | POS | 0,821 | POS | POS | | |
| 18 | PE | | POS | | | POS | POS | POS | 0,734 | POS | POS | | |
| 19 | PE | | POS | | | POS | POS | POS | 0,683 | POS | POS | | |
| 20 | PE | | POS | | | POS | NEG | POS | 0,511 | POS | POS | | |
| 26 | PE | | POS | | | POS | NEG | POS | 0,787 | POS | POS | | |
| 27 | PE | | POS | | | POS | NEG | NEG | 0,280 | POS | POS | | |
| 30 | PE | | POS | | | POS | POS | POS | 0,316 | POS | NEG | | |
| 32 | PE | | POS | | | POS | NEG | NEG | 0,215 | POS | NEG | | |
| 33 | PE | | POS | | | POS | NEG | POS | 0,998 | POS | POS | | |
| 45 | PE | | POS | | | POS | NEG | POS | 0,489 | POS | POS | | |
| 380 | SP | POS | | | | | POS | POS | 0,948 | POS | POS | | |
| 393 | SP | POS | | | | | POS | NEG | 0,167 | POS | NEG | | |
| 413 | SP | POS | | | | | POS | POS | 0,792 | POS | POS | | |
| 447 | SP | POS | | | | | POS | POS | 0,781 | POS | POS | | |
| 501 | SP | POS | | | | | POS | POS | 1,008 | POS | POS | | |
| 636 | SP | POS | | | | | POS | POS | 0,977 | POS | POS | | |
| 655 | SP | POS | | | | | POS | POS | 0,976 | POS | POS | | |
| 689 | SP | POS | | | | | POS | POS | 1,026 | POS | POS | | |

| ID | Brazilian State | Parasitological | ELISA SLA | ELISA rK39 | RDT rK39 | IFI | PCR | ELISA Q5 | D.O | DPP - LVC | RDT Q5/2019 | RDT Q5/2017 | RDT Q5/2021 |
|---|---|---|---|---|---|---|---|---|---|---|---|---|---|
| 775 | SP | POS | | | | | POS | POS | 0,722 | POS | POS | | |
| 1048 | SP | POS | | | | | POS | POS | 1,005 | POS | POS | | |
| 1078 | SP | POS | | | | | POS | POS | 0,938 | POS | POS | | |
| 1085 | SP | POS | | | | | POS | POS | 0,935 | POS | POS | | |
| 1090 | SP | POS | | | | | POS | POS | 0,740 | POS | POS | | |
| 1093 | SP | POS | | | | | POS | POS | 0,822 | POS | POS | | |
| 1122 | SP | POS | | | | | POS | POS | 1,033 | POS | POS | | |
| 1227 | SP | POS | | | | | POS | POS | 0,989 | POS | POS | | |
| 1255 | SP | POS | | | | | POS | POS | 0,864 | POS | POS | | |
| 1337 | SP | POS | | | | | POS | POS | 0,850 | POS | POS | | |
| 1414 | SP | POS | | | | | POS | POS | 2,237 | POS | POS | | |
| 1426 | SP | POS | | | | | POS | NEG | 0,101 | POS | NEG | | |
| 1476 | SP | POS | | | | | POS | POS | 0,878 | POS | POS | | |
| 1480 | SP | POS | | | | | POS | POS | 0,685 | POS | POS | | |
| 1575 | SP | POS | | | | | POS | POS | 0,730 | POS | POS | | |
| 1 | SP | POS | | | | | POS | POS | 1,835 | POS | POS | | |
| 2 | SP | POS | | | | | POS | POS | 0,567 | POS | POS | | |
| 3 | SP | POS | | | | | POS | POS | 1,193 | POS | POS | | |
| 4 | SP | POS | | | | | POS | POS | 1,397 | POS | POS | | |
| 5 | SP | POS | | | | | POS | NEG | 0,286 | POS | NEG | | |
| 6 | SP | POS | | | | | POS | POS | 0,225 | POS | NEG | | |
| 7 | SP | POS | | | | | POS | POS | 0,730 | POS | POS | | |
| 8 | SP | POS | | | | | POS | POS | 0,349 | POS | POS | | |
| 9 | SP | POS | | | | | POS | POS | 0,700 | POS | POS | | |
| 10 | SP | POS | | | | | POS | POS | 0,495 | POS | POS | | |
| 17 | SP | POS | | | | | POS | POS | 1,589 | POS | POS | | |
| 18 | SP | POS | | | | | POS | POS | 0,631 | POS | NEG | | |
| 20 | SP | POS | | | | | POS | POS | 0,724 | POS | POS | | |
| 21 | SP | POS | | | | | POS | POS | 0,638 | POS | POS | | |
| 23 | SP | POS | | | | | POS | POS | 0,713 | POS | POS | | |
| 24 | SP | POS | | | | | POS | POS | 1,026 | POS | POS | | |
| 33 | SP | POS | | | | | POS | POS | 0,976 | POS | POS | | |
| 50 | SP | POS | | | | | POS | POS | 1,008 | POS | POS | | |
| 66 | SP | POS | | | | | POS | POS | 0,977 | POS | POS | | |
| 106 | SP | POS | | | | | POS | POS | 0,948 | POS | POS | | |
| 112 | SP | POS | | | | | POS | POS | 0,792 | POS | POS | | |
| 136 | SP | POS | | | | | POS | POS | 0,781 | POS | POS | | |
| 137 | SP | POS | | | | | POS | NEG | 0,167 | POS | NEG | | |
| 151 | SP | POS | | | | | POS | POS | 1,033 | POS | POS | | |
| 174 | SP | POS | | | | | POS | POS | 0,989 | POS | POS | | |

| ID | Brazilian State | ELISA SLA | ELISA Q5 | D.O | RDT Q5/2019 | RDT Q5/2017 | RDT Q5/2021 | DPP - LVC | | |
|---|---|---|---|---|---|---|---|---|---|---|
| 3 | PE | NEG | NEG | 0,126 | NEG | | | NEG | | |
| 5 | PE | NEG | NEG | 0,056 | NEG | | | NEG | | |
| 6 | PE | NEG | **POS** | **0,636** | **POS** | | | NEG | MN | 0,094 |
| 7 | PE | NEG | NEG | 0,051 | NEG | | | NEG | DP | 0,096 |
| 8 | PE | NEG | NEG | 0,063 | NEG | | | NEG | Cut-off | 0,286 |
| 9 | PE | NEG | NEG | 0,053 | NEG | | | NEG | | |
| 10 | PE | NEG | NEG | 0,014 | NEG | | | NEG | | |
| 14 | PE | NEG | NEG | 0,011 | NEG | | | NEG | | |
| 21 | PE | NEG | NEG | 0,083 | NEG | | | NEG | | |
| 22 | PE | NEG | NEG | 0,037 | NEG | | | NEG | | |
| 23 | PE | NEG | NEG | 0,099 | NEG | | | NEG | | |
| 24 | PE | NEG | NEG | 0,066 | NEG | | | NEG | | |
| 25 | PE | NEG | NEG | 0,033 | NEG | | | NEG | | |
| 28 | PE | NEG | NEG | 0,139 | NEG | | | NEG | | |
| 29 | PE | NEG | NEG | 0,148 | NEG | | | NEG | | |
| 34 | PE | NEG | NEG | 0,096 | NEG | | | NEG | | |
| 36 | PE | NEG | NEG | 0,007 | NEG | | | NEG | | |
| 40 | PE | NEG | NEG | 0,255 | NEG | | | NEG | | |
| 42 | PE | NEG | NEG | 0,112 | NEG | | | NEG | | |
| 44 | PE | NEG | NEG | 0,056 | NEG | | | NEG | | |
| 47 | PE | NEG | NEG | 0,053 | NEG | | | NEG | | |
| 48 | PE | NEG | NEG | 0,030 | NEG | | | NEG | | |
| 49 | PE | NEG | NEG | 0,163 | NEG | | | NEG | | |
| 50 | PE | NEG | NEG | 0,103 | NEG | | | NEG | | |
| 52 | PE | NEG | NEG | 0,172 | NEG | | | NEG | | |
| 53 | PE | NEG | NEG | 0,136 | NEG | | | NEG | | |
| 54 | PE | NEG | NEG | 0,071 | NEG | | | NEG | | |
| 60 | PE | NEG | NEG | 0,076 | NEG | | | NEG | | |
| 61 | PE | NEG | NEG | 0,224 | NEG | | | NEG | | |
| 62 | PE | NEG | NEG | 0,127 | **POS** | | | NEG | | |
| BOB | BA | NEG | NEG | 0,072 | NEG | NEG | NEG | NEG | | |
| DAIANA | BA | NEG | NEG | 0,044 | NEG | NEG | NEG | NEG | | |
| BELA | BA | NEG | NEG | 0,114 | NEG | NEG | NEG | NEG | | |
| LUA | BA | NEG | NEG | 0,091 | NEG | NEG | NEG | NEG | | |
| SERENA | BA | NEG | NEG | 0,035 | NEG | NEG | NEG | NEG | | |
| LULU | BA | NEG | NEG | 0,052 | NEG | NEG | NEG | NEG | | |
| RICKY | BA | NEG | NEG | 0,071 | NEG | NEG | NEG | NEG | | |
| BIA | BA | NEG | NEG | 0,107 | NEG | NEG | NEG | NEG | | |
| TITA | BA | NEG | NEG | 0,048 | NEG | NEG | NEG | NEG | | |
| LOPO | BA | NEG | NEG | 0,072 | NEG | NEG | NEG | NEG | | |
| TOM | BA | NEG | NEG | 0,026 | NEG | NEG | NEG | NEG | | |
| CAZUZA | BA | NEG | NEG | 0,079 | NEG | NEG | NEG | NEG | | |
| CLOTILDE | BA | NEG | NEG | 0,032 | NEG | | NEG | NEG | | |
| PEU | BA | NEG | NEG | 0,081 | NEG | | NEG | NEG | | |
| BRISA | BA | NEG | NEG | 0,051 | NEG | | NEG | NEG | | |
| media | BA | NEG | NEG | 0,106 | NEG | | NEG | NEG | | |
| devpad | BA | NEG | NEG | 0,060 | NEG | | NEG | NEG | | |

ID: identification; SLA: soluble *Leishmania* antigen; RDT: rapid diagnostic test; IFI: indirect immunofluorescence; PCR: polymerase chain reaction; DAT: direct agglutination test; D.O: optical density; MN: average of negatives; DP: standard deviation; MS: Mato Grosso do Sul; PE: Pernambuco; PI: Piauí; BA: Bahia; SP: São Paulo; IDMR: intradermal Montenegro reaction; DPP-LVC: dual-path platform; ELISA: enzyme immunoassay; POS: positive; NEG: negative.
